# Poly[di-μ_9_-citrato-tetra­sodiumzinc]

**DOI:** 10.1107/S1600536813030067

**Published:** 2013-11-20

**Authors:** Yu-Hong Ma, Hong-Wei Yang, Jing-Tuan Hao, Pi-Zhuang Ma, Ting Yao

**Affiliations:** a Air Force Service College, Xuzhou 221000, People’s Republic of China; b Logistics College, Beijing 100858, People’s Republic of China

## Abstract

In the title compound, [Na_4_Zn(C_6_H_5_O_7_)_2_]_*n*_, the Zn^II^ ion lies on an inversion center and is coordinated by six O atoms from two citrate ligands, forming a distorted octa­hedral geometry. There are two crystallographically independent Na^+^ cations in the asymmetric unit. One Na^+^ cation exhibits a distorted square-pyramidal geometry defined by five O atoms from four citrate ligands. The other Na^+^ cation is surrounded by six O atoms from five citrate ligands in a distorted octa­hedral geometry. The Na^+^ cations are bridged by citrate carboxyl­ate groups, forming a layer parallel to (100). The layers are further assembled into a three-dimensional network with the [Zn(citrate)_2_]^4−^ building units as ‘pillars’; O—H⋯O hydrogen bonds also stabilize the structure.

## Related literature

For an isotypic compound, see: Liu *et al.* (2012 ▶).
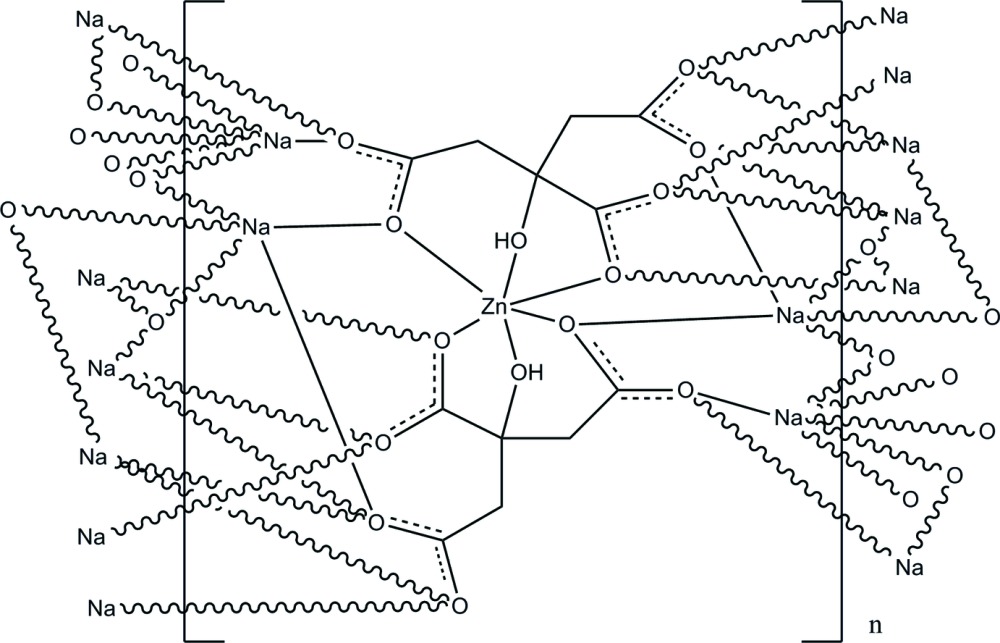


## Experimental

### 

#### Crystal data

[Na_4_Zn(C_6_H_5_O_7_)_2_]
*M* *_r_* = 535.55
Monoclinic, 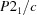

*a* = 7.9642 (16) Å
*b* = 12.530 (3) Å
*c* = 8.7090 (17) Å
β = 113.66 (3)°
*V* = 796.0 (3) Å^3^
*Z* = 2
Mo *K*α radiation
μ = 1.74 mm^−1^
*T* = 293 K
0.21 × 0.21 × 0.20 mm

#### Data collection

Rigaku SCXmini CCD diffractometer
Absorption correction: multi-scan (*ABSCOR*; Higashi, 1995 ▶) *T*_min_ = 0.712, *T*_max_ = 0.722
8270 measured reflections
1831 independent reflections
1570 reflections with *I* > 2σ(*I*)
*R*_int_ = 0.048

#### Refinement

*R*[*F* ^2^ > 2σ(*F* ^2^)] = 0.037
*wR*(*F* ^2^) = 0.075
*S* = 1.15
1831 reflections
145 parameters
H atoms treated by a mixture of independent and constrained refinement
Δρ_max_ = 0.37 e Å^−3^
Δρ_min_ = −0.47 e Å^−3^

### 

Data collection: *CrystalClear* (Rigaku, 2005 ▶); cell refinement: *CrystalClear*; data reduction: *CrystalClear*; program(s) used to solve structure: *SHELXS97* (Sheldrick, 2008 ▶); program(s) used to refine structure: *SHELXL97* (Sheldrick, 2008 ▶); molecular graphics: *XP* in *SHELXTL* (Sheldrick, 2008 ▶) and *DIAMOND* (Brandenburg, 1999 ▶); software used to prepare material for publication: *SHELXTL*.

## Supplementary Material

Crystal structure: contains datablock(s) I. DOI: 10.1107/S1600536813030067/hy2639sup1.cif

Structure factors: contains datablock(s) I. DOI: 10.1107/S1600536813030067/hy2639Isup2.hkl

Additional supplementary materials:  crystallographic information; 3D view; checkCIF report

## Figures and Tables

**Table 1:** Hydrogen-bond geometry (Å, °)

*D*—H⋯*A*	*D*—H	H⋯*A*	*D*⋯*A*	*D*—H⋯*A*
O7—H1⋯O2^i^	0.95 (3)	1.69 (3)	2.635 (2)	174 (3)

Symmetry code: (i) 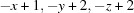.

**Fig. 1.**
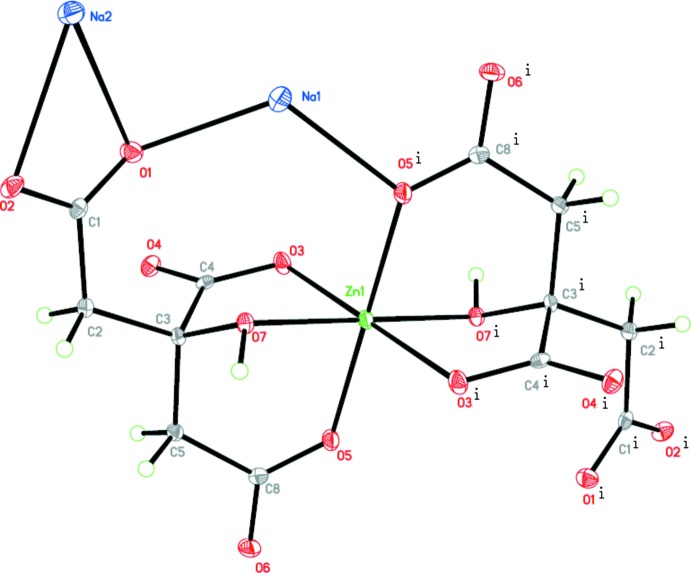
The asymmetric unit of the title compound, showing the 30% probability displacement ellipsoids. [Symmetry code: (i) 1-x, 2-y, 1-z.]

**Fig. 2.**
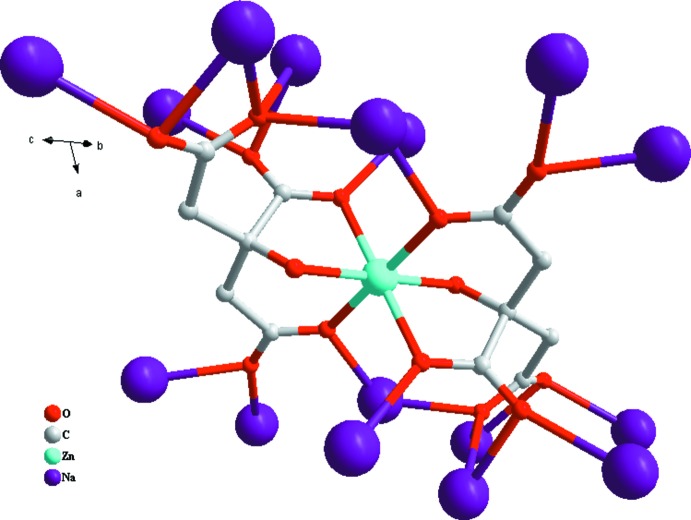
The [Zn(C6H5O7)2]4- building unit bridges sixteen Na+ cations.

**Fig. 3.**
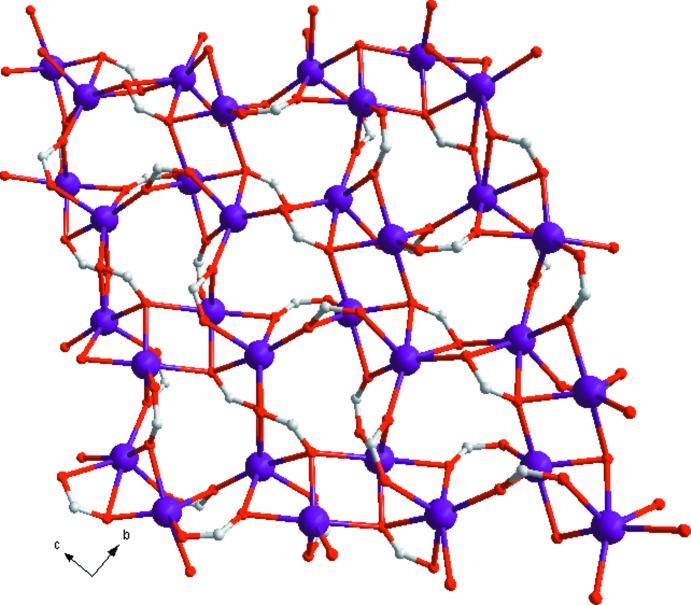
A view of the two-dimensional layer in the bc plane.

**Fig. 4.**
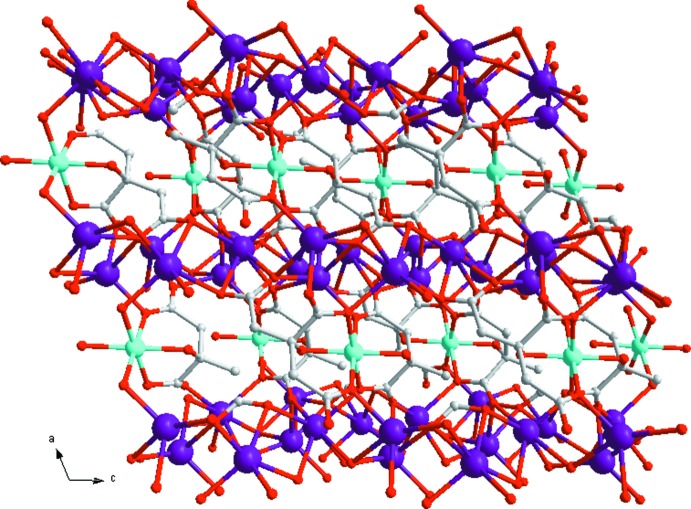
A view of the three-dimensional network in the title compound.

## supplementary crystallographic information

### 1. Comment

Citric acid has been widely used for the construction of coordination polymers due to their diverse coordination modes (Liu *et al.*, 2012). Here, we report a new three-dimensional coordination polymer, [Na_4_Zn(C_6_H_5_O_7_)_2_]_n_, based on citric acid.

As shown in Fig. 1, the asymmetric unit of the title compound consists of half a Zn^II^ ion, two Na^+^ cations and a citrate anion. The Zn^II^ ion lies on a crystallographic inversion center and is coordinated by six O atoms from two different citrate ligands, forming a distorted octahedral geometry. Three O atoms of each citrate ligand are bonded to the Zn^II^ ion, one of which is the hydroxy O atom and the other two are from different carboxylate groups. Thus, two citrate ligands and one Zn^II^ ion form a [Zn(C_6_H_5_O_7_)_2_]^4-^ building unit. This unit bridges sixteen Na^+^ cations (Fig. 2). Na1 exhibits a distorted square-pyramidal geometry, defined by five O atoms from four different citrate ligands. Na2 is surrounded by six O atoms from five different citrate ligands, building a distorted octahedral geometry. The Na^+^ cations are bridged by carboxylate groups from the citrate ligands into a two-dimensional layer parallel to (100) (Fig. 3). The layers are further assembled into a three-dimensional network through [Zn(C_6_H_5_O_7_)_2_]^4-^ building units as 'pillars' (Fig. 4).

### 2. Experimental

A mixture of citric acid (0.2 mmol), NaOH (0.2 mmol) and zinc nitrate hexahydrate (0.1 mmol) was dissolved in DMAC/H_2_O solvent (5 ml, v/v = 1:4) (DMAC = *N*,*N*'-dimethylacetamide) and placed in a capped vial (10 ml), which was heated to 363 K for three days and then cooled to room temperature. The crystals obtained were washed with water and dried in air.

### 3. Refinement

C-bound H atoms were placed at calculated positions and refined as riding atoms, with C—H = 0.97 Å and with *U*_iso_(H) = 1.2*U*_eq_(C). The hydroxy H atom was located in a difference map and refined isotropically.

### Crystal data

**Table:**

[Na_4_Zn(C_6_H_5_O_7_)_2_]	*F*(000) = 536
*M**_r_* = 535.55	*D*_x_ = 2.234 Mg m^−^^3^
Monoclinic, *P*2_1_/*c*	Mo *K*α radiation, λ = 0.71073 Å
Hall symbol: -P 2ybc	Cell parameters from 7740 reflections
*a* = 7.9642 (16) Å	θ = 3.0–27.5°
*b* = 12.530 (3) Å	µ = 1.74 mm^−^^1^
*c* = 8.7090 (17) Å	*T* = 293 K
β = 113.66 (3)°	Block, colorless
*V* = 796.0 (3) Å^3^	0.21 × 0.21 × 0.20 mm
*Z* = 2	

### Data collection

**Table:**

Rigaku SCXmini CCD diffractometer	1831 independent reflections
Radiation source: fine-focus sealed tube	1570 reflections with *I* > 2σ(*I*)
Graphite monochromator	*R*_int_ = 0.048
ω scan	θ_max_ = 27.5°, θ_min_ = 3.0°
Absorption correction: multi-scan (*ABSCOR*; Higashi, 1995)	*h* = −10→10
*T*_min_ = 0.712, *T*_max_ = 0.722	*k* = −16→16
8270 measured reflections	*l* = −11→11

### Refinement

**Table:**

Refinement on *F*^2^	Primary atom site location: structure-invariant direct methods
Least-squares matrix: full	Secondary atom site location: difference Fourier map
*R*[*F*^2^ > 2σ(*F*^2^)] = 0.037	Hydrogen site location: inferred from neighbouring sites
*wR*(*F*^2^) = 0.075	H atoms treated by a mixture of independent and constrained refinement
*S* = 1.15	*w* = 1/[σ^2^(*F*_o_^2^) + (0.0289*P*)^2^ + 0.3351*P*] where *P* = (*F*_o_^2^ + 2*F*_c_^2^)/3
1831 reflections	(Δ/σ)_max_ = 0.001
145 parameters	Δρ_max_ = 0.37 e Å^−^^3^
0 restraints	Δρ_min_ = −0.47 e Å^−^^3^

### Special details

**Table:**

Geometry. All e.s.d.'s (except the e.s.d. in the dihedral angle between two l.s. planes) are estimated using the full covariance matrix. The cell e.s.d.'s are taken into account individually in the estimation of e.s.d.'s in distances, angles and torsion angles; correlations between e.s.d.'s in cell parameters are only used when they are defined by crystal symmetry. An approximate (isotropic) treatment of cell e.s.d.'s is used for estimating e.s.d.'s involving l.s. planes.
Refinement. Refinement of *F*^2^ against ALL reflections. The weighted *R*-factor *wR* and goodness of fit *S* are based on *F*^2^, conventional *R*-factors *R* are based on *F*, with *F* set to zero for negative *F*^2^. The threshold expression of *F*^2^ > σ(*F*^2^) is used only for calculating *R*-factors(gt) *etc*. and is not relevant to the choice of reflections for refinement. *R*-factors based on *F*^2^ are statistically about twice as large as those based on *F*, and *R*- factors based on ALL data will be even larger.

### Fractional atomic coordinates and isotropic or equivalent isotropic displacement parameters (Å^2^)

**Table:**

	*x*	*y*	*z*	*U*_iso_*/*U*_eq_	
O7	0.4930 (2)	0.98761 (13)	0.7363 (2)	0.0127 (4)	
O3	0.2766 (2)	0.89905 (13)	0.4482 (2)	0.0178 (4)	
O4	0.1751 (2)	0.77628 (13)	0.5749 (2)	0.0175 (4)	
O6	0.8225 (3)	0.72885 (15)	0.7149 (2)	0.0233 (4)	
O5	0.6783 (2)	0.87114 (13)	0.5716 (2)	0.0175 (4)	
C8	0.7041 (3)	0.8007 (2)	0.6829 (3)	0.0141 (5)	
C4	0.2806 (3)	0.84956 (19)	0.5774 (3)	0.0124 (5)	
C5	0.5911 (3)	0.80021 (19)	0.7885 (3)	0.0132 (5)	
H5A	0.6748	0.8118	0.9042	0.016*	
H5B	0.5407	0.7291	0.7822	0.016*	
C3	0.4328 (3)	0.87982 (18)	0.7479 (3)	0.0118 (5)	
C2	0.3615 (3)	0.87711 (19)	0.8861 (3)	0.0144 (5)	
H2A	0.3180	0.8056	0.8920	0.017*	
H2B	0.4629	0.8916	0.9923	0.017*	
O2	0.1969 (2)	0.98895 (14)	0.9984 (2)	0.0179 (4)	
Zn1	0.5000	1.0000	0.5000	0.01321 (12)	
Na2	−0.05408 (14)	1.12137 (8)	0.83937 (13)	0.0216 (3)	
Na1	0.10230 (14)	1.11830 (8)	0.53517 (13)	0.0222 (3)	
O1	0.1002 (2)	0.98321 (13)	0.7212 (2)	0.0186 (4)	
C1	0.2085 (3)	0.95534 (19)	0.8644 (3)	0.0131 (5)	
H1	0.606 (4)	1.000 (2)	0.828 (4)	0.016*	

### Atomic displacement parameters (Å^2^)

**Table:**

	*U*^11^	*U*^22^	*U*^33^	*U*^12^	*U*^13^	*U*^23^
O7	0.0154 (9)	0.0111 (9)	0.0118 (9)	−0.0021 (7)	0.0057 (7)	−0.0007 (7)
O3	0.0185 (9)	0.0207 (10)	0.0114 (9)	−0.0038 (8)	0.0030 (8)	0.0027 (7)
O4	0.0173 (9)	0.0160 (9)	0.0210 (10)	−0.0047 (7)	0.0095 (8)	−0.0030 (7)
O6	0.0225 (10)	0.0256 (11)	0.0245 (11)	0.0128 (8)	0.0123 (9)	0.0071 (8)
O5	0.0223 (10)	0.0168 (9)	0.0180 (9)	0.0037 (7)	0.0127 (8)	0.0041 (7)
C8	0.0123 (12)	0.0156 (13)	0.0137 (12)	0.0008 (10)	0.0045 (10)	−0.0014 (10)
C4	0.0110 (12)	0.0132 (12)	0.0151 (12)	0.0031 (9)	0.0072 (10)	−0.0014 (9)
C5	0.0145 (12)	0.0127 (12)	0.0131 (12)	0.0023 (10)	0.0065 (10)	0.0017 (9)
C3	0.0136 (12)	0.0104 (11)	0.0123 (12)	−0.0003 (9)	0.0062 (10)	0.0028 (9)
C2	0.0155 (12)	0.0160 (13)	0.0134 (13)	0.0014 (10)	0.0076 (11)	0.0024 (10)
O2	0.0176 (9)	0.0229 (10)	0.0162 (9)	0.0018 (8)	0.0098 (8)	−0.0047 (7)
Zn1	0.0152 (2)	0.0130 (2)	0.0124 (2)	0.00022 (17)	0.00663 (17)	0.00209 (16)
Na2	0.0197 (5)	0.0236 (6)	0.0216 (6)	0.0023 (4)	0.0084 (5)	−0.0013 (4)
Na1	0.0208 (6)	0.0253 (6)	0.0223 (6)	−0.0022 (4)	0.0105 (5)	−0.0007 (4)
O1	0.0178 (9)	0.0208 (10)	0.0159 (9)	0.0024 (7)	0.0053 (8)	0.0006 (7)
C1	0.0134 (12)	0.0113 (11)	0.0165 (13)	−0.0039 (10)	0.0080 (11)	−0.0011 (10)

### Geometric parameters (Å, º)

**Table:**

O7—C3	1.450 (3)	O2—Na2	2.539 (2)
O7—Zn1	2.0866 (17)	Zn1—O5^iv^	2.0742 (17)
O3—C4	1.274 (3)	Zn1—O3^iv^	2.0796 (17)
O3—Zn1	2.0796 (17)	Zn1—O7^iv^	2.0866 (17)
O3—Na2^i^	2.432 (2)	Na2—O4^v^	2.415 (2)
O4—C4	1.239 (3)	Na2—O3^i^	2.432 (2)
O4—Na2^ii^	2.415 (2)	Na2—O6^vi^	2.478 (2)
O4—Na1^i^	2.417 (2)	Na2—O2^vii^	2.548 (2)
O6—C8	1.251 (3)	Na2—O1	2.565 (2)
O6—Na1^iii^	2.443 (2)	Na1—O5^iv^	2.288 (2)
O6—Na2^iii^	2.478 (2)	Na1—O1	2.348 (2)
O5—C8	1.266 (3)	Na1—O4^i^	2.417 (2)
O5—Zn1	2.0742 (17)	Na1—O6^vi^	2.443 (2)
O5—Na1^iv^	2.288 (2)	Na1—O1^i^	2.512 (2)
C8—C5	1.523 (3)	Na1—C4^i^	2.833 (3)
C8—Na1^iv^	3.061 (3)	Na1—C8^iv^	3.061 (3)
C4—C3	1.540 (3)	O1—C1	1.248 (3)
C4—Na1^i^	2.833 (3)	O7—H1	0.95 (3)
C5—C3	1.534 (3)	C2—H2A	0.97
C3—C2	1.523 (3)	C2—H2B	0.97
C2—C1	1.516 (3)	C5—H5A	0.97
O2—C1	1.279 (3)	C5—H5B	0.97
			
C3—O7—Zn1	106.16 (13)	Na1—Na2—Na1^viii^	102.34 (4)
C4—O3—Zn1	112.86 (16)	O4^v^—Na2—Na2^vii^	113.39 (6)
C4—O3—Na2^i^	127.44 (16)	O3^i^—Na2—Na2^vii^	120.36 (6)
Zn1—O3—Na2^i^	119.59 (8)	O6^vi^—Na2—Na2^vii^	125.52 (7)
C4—O4—Na2^ii^	160.44 (17)	O2—Na2—Na2^vii^	38.48 (4)
C4—O4—Na1^i^	96.18 (14)	O2^vii^—Na2—Na2^vii^	38.32 (4)
Na2^ii^—O4—Na1^i^	98.47 (7)	O1—Na2—Na2^vii^	76.31 (5)
C8—O6—Na1^iii^	119.97 (16)	Na1—Na2—Na2^vii^	120.15 (4)
C8—O6—Na2^iii^	154.24 (17)	Na1^viii^—Na2—Na2^vii^	114.57 (5)
Na1^iii^—O6—Na2^iii^	85.80 (7)	O5^iv^—Na1—O1	122.68 (8)
C8—O5—Zn1	130.84 (16)	O5^iv^—Na1—O4^i^	122.16 (7)
C8—O5—Na1^iv^	115.86 (16)	O1—Na1—O4^i^	114.15 (7)
Zn1—O5—Na1^iv^	112.04 (8)	O5^iv^—Na1—O6^vi^	112.02 (8)
O6—C8—O5	123.2 (2)	O1—Na1—O6^vi^	82.02 (7)
O6—C8—C5	116.0 (2)	O4^i^—Na1—O6^vi^	84.37 (8)
O5—C8—C5	120.8 (2)	O5^iv^—Na1—O1^i^	89.47 (7)
O4—C4—O3	124.7 (2)	O1—Na1—O1^i^	93.91 (7)
O4—C4—C3	117.7 (2)	O4^i^—Na1—O1^i^	76.45 (7)
O3—C4—C3	117.6 (2)	O6^vi^—Na1—O1^i^	156.79 (8)
C8—C5—C3	119.2 (2)	O5^iv^—Na1—C4^i^	137.95 (8)
O7—C3—C2	108.32 (19)	O1—Na1—C4^i^	92.09 (8)
O7—C3—C5	110.93 (19)	O4^i^—Na1—C4^i^	25.78 (6)
C2—C3—C5	109.63 (19)	O6^vi^—Na1—C4^i^	94.17 (8)
O7—C3—C4	108.45 (18)	O1^i^—Na1—C4^i^	63.03 (7)
C2—C3—C4	110.9 (2)	O5^iv^—Na1—C8^iv^	21.85 (6)
C5—C3—C4	108.56 (19)	O1—Na1—C8^iv^	144.32 (8)
C1—C2—C3	115.0 (2)	O4^i^—Na1—C8^iv^	100.39 (7)
C1—O2—Na2	92.67 (15)	O6^vi^—Na1—C8^iv^	111.12 (7)
C1—O2—Na2^vii^	122.64 (15)	O1^i^—Na1—C8^iv^	85.42 (7)
Na2—O2—Na2^vii^	103.20 (7)	C4^i^—Na1—C8^iv^	118.60 (8)
O5^iv^—Zn1—O5	180.0	O5^iv^—Na1—Na1^i^	112.02 (7)
O5^iv^—Zn1—O3^iv^	90.85 (7)	O1—Na1—Na1^i^	49.04 (5)
O5—Zn1—O3^iv^	89.15 (7)	O4^i^—Na1—Na1^i^	96.43 (6)
O5^iv^—Zn1—O3	89.15 (7)	O6^vi^—Na1—Na1^i^	126.69 (7)
O5—Zn1—O3	90.85 (7)	O1^i^—Na1—Na1^i^	44.88 (5)
O3^iv^—Zn1—O3	180.000 (1)	C4^i^—Na1—Na1^i^	71.49 (6)
O5^iv^—Zn1—O7^iv^	86.09 (7)	C8^iv^—Na1—Na1^i^	120.93 (7)
O5—Zn1—O7^iv^	93.91 (7)	O5^iv^—Na1—Na2	155.15 (7)
O3^iv^—Zn1—O7^iv^	79.02 (7)	O1—Na1—Na2	49.81 (5)
O3—Zn1—O7^iv^	100.98 (7)	O4^i^—Na1—Na2	74.74 (5)
O5^iv^—Zn1—O7	93.91 (7)	O6^vi^—Na1—Na2	47.54 (5)
O5—Zn1—O7	86.09 (7)	O1^i^—Na1—Na2	113.50 (6)
O3^iv^—Zn1—O7	100.98 (7)	C4^i^—Na1—Na2	65.27 (6)
O3—Zn1—O7	79.02 (7)	C8^iv^—Na1—Na2	157.96 (6)
O7^iv^—Zn1—O7	180.000 (1)	Na1^i^—Na1—Na2	81.11 (4)
O5^iv^—Zn1—Na1	35.88 (5)	O5^iv^—Na1—Zn1	32.08 (5)
O5—Zn1—Na1	144.12 (5)	O1—Na1—Zn1	90.68 (6)
O3^iv^—Zn1—Na1	115.73 (5)	O4^i^—Na1—Zn1	151.83 (6)
O3—Zn1—Na1	64.27 (5)	O6^vi^—Na1—Zn1	113.43 (6)
O7^iv^—Zn1—Na1	115.05 (6)	O1^i^—Na1—Zn1	89.37 (5)
O7—Zn1—Na1	64.95 (6)	C4^i^—Na1—Zn1	152.38 (6)
O5^iv^—Zn1—Na1^iv^	144.12 (5)	C8^iv^—Na1—Zn1	53.66 (5)
O5—Zn1—Na1^iv^	35.88 (5)	Na1^i^—Na1—Zn1	90.01 (4)
O3^iv^—Zn1—Na1^iv^	64.27 (5)	Na2—Na1—Zn1	133.42 (4)
O3—Zn1—Na1^iv^	115.73 (5)	O5^iv^—Na1—Na2^ix^	86.09 (5)
O7^iv^—Zn1—Na1^iv^	64.95 (6)	O1—Na1—Na2^ix^	150.32 (6)
O7—Zn1—Na1^iv^	115.05 (6)	O4^i^—Na1—Na2^ix^	40.74 (5)
Na1—Zn1—Na1^iv^	180.0	O6^vi^—Na1—Na2^ix^	79.96 (6)
O4^v^—Na2—O3^i^	100.76 (7)	O1^i^—Na1—Na2^ix^	93.45 (6)
O4^v^—Na2—O6^vi^	92.41 (7)	C4^i^—Na1—Na2^ix^	66.06 (6)
O3^i^—Na2—O6^vi^	98.70 (7)	C8^iv^—Na1—Na2^ix^	64.99 (5)
O4^v^—Na2—O2	132.94 (7)	Na1^i^—Na1—Na2^ix^	131.30 (6)
O3^i^—Na2—O2	125.58 (7)	Na2—Na1—Na2^ix^	101.14 (3)
O6^vi^—Na2—O2	88.62 (7)	Zn1—Na1—Na2^ix^	118.12 (3)
O4^v^—Na2—O2^vii^	86.70 (7)	C1—O1—Na1	134.25 (16)
O3^i^—Na2—O2^vii^	102.18 (7)	C1—O1—Na1^i^	133.31 (16)
O6^vi^—Na2—O2^vii^	158.90 (8)	Na1—O1—Na1^i^	86.09 (7)
O2—Na2—O2^vii^	76.80 (7)	C1—O1—Na2	92.17 (15)
O4^v^—Na2—O1	168.90 (7)	Na1—O1—Na2	85.84 (6)
O3^i^—Na2—O1	77.54 (7)	Na1^i^—O1—Na2	117.27 (8)
O6^vi^—Na2—O1	77.12 (7)	O1—C1—O2	123.1 (2)
O2—Na2—O1	51.60 (6)	O1—C1—C2	120.3 (2)
O2^vii^—Na2—O1	104.40 (7)	O2—C1—C2	116.6 (2)
O4^v^—Na2—Na1	125.09 (6)	Zn1—O7—H1	115 (2)
O3^i^—Na2—Na1	62.16 (5)	C3—O7—H1	108.9 (16)
O6^vi^—Na2—Na1	46.67 (5)	C1—C2—H2A	109
O2—Na2—Na1	87.87 (5)	C1—C2—H2B	109
O2^vii^—Na2—Na1	145.37 (6)	C3—C2—H2A	108
O1—Na2—Na1	44.35 (5)	C3—C2—H2B	109
O4^v^—Na2—Na1^viii^	40.79 (5)	H2A—C2—H2B	108
O3^i^—Na2—Na1^viii^	123.00 (6)	C3—C5—H5A	107
O6^vi^—Na2—Na1^viii^	57.61 (5)	C3—C5—H5B	108
O2—Na2—Na1^viii^	106.39 (6)	C8—C5—H5A	107
O2^vii^—Na2—Na1^viii^	111.68 (5)	C8—C5—H5B	108
O1—Na2—Na1^viii^	131.31 (6)	H5A—C5—H5B	107

Symmetry codes: (i) −*x*, −*y*+2, −*z*+1; (ii) −*x*, *y*−1/2, −*z*+3/2; (iii) −*x*+1, *y*−1/2, −*z*+3/2; (iv) −*x*+1, −*y*+2, −*z*+1; (v) −*x*, *y*+1/2, −*z*+3/2; (vi) −*x*+1, *y*+1/2, −*z*+3/2; (vii) −*x*, −*y*+2, −*z*+2; (viii) *x*, −*y*+5/2, *z*+1/2; (ix) *x*, −*y*+5/2, *z*−1/2.

### Hydrogen-bond geometry (Å, º)

**Table:**

*D*—H···*A*	*D*—H	H···*A*	*D*···*A*	*D*—H···*A*
O7—H1···O2^x^	0.95 (3)	1.69 (3)	2.635 (2)	174 (3)

Symmetry code: (x) −*x*+1, −*y*+2, −*z*+2.
